# Optimization and comparison of genomic DNA extraction from whole blood collected in PAXgene blood RNA tube using automated platforms

**DOI:** 10.1515/cclm-2025-1079

**Published:** 2025-10-20

**Authors:** Jianlan You, Tomoe Shiomi, Paul Zappile, Luis Chiriboga, Sandra Mendoza, Andre L. Moreira

**Affiliations:** Center for Biospecimen Research & Development, 12296 New York University Grossman School of Medicine, New York, NY, USA; Genome Technology Center, New York University Grossman School of Medicine, New York, NY, USA; Department of Pathology, New York University Grossman School of Medicine, New York, NY, USA

**Keywords:** automated extraction, genomic DNA extraction, high-throughput extraction, magnetic bead-based extraction, PAXgene blood RNA tube

## Abstract

**Objectives:**

With the development of genomic technologies, the isolation of genomic DNA (gDNA) from clinical samples has become more important for both clinical diagnostics and research studies. Blood samples collected in PAXgene Blood RNA Tubes, which are typically used for RNA extraction, can be used for gDNA extraction, particularly in clinical studies when only such blood samples are available.

**Methods:**

We optimized the pre-treatment of blood samples collected in PAXgene Blood RNA Tubes. For the first time, three magnetic bead-based automated platforms (QIAsymphony SP, Maxwell RSC and KingFisher Apex) were compared for gDNA extraction from these blood samples. Additionally, the effects of storage at 4 °C or freeze-thaw cycles of the blood samples on gDNA yield were investigated. High-throughput extraction in 96-well format was evaluated.

**Results:**

Systematic optimization of blood sample pre-treatment shows that prolonged incubation at room temperature and/or increased centrifugation speed and time improved gDNA yield from blood samples collected in PAXgene Blood RNA Tubes. QIAsymphony SP (4.27 ± 2.19 µg) and Maxwell RSC (4.82 ± 2.96 µg) produced significantly higher gDNA yields than KingFisher Apex (1.09 ± 0.61 µg). Higher gDNA yields were obtained with shorter storage time at 4 °C or fewer freeze-thaw cycles of the blood samples. In the 96-well format extraction, gDNA yields ranged from 0.24 to 13.46 µg.

**Conclusions:**

Not all magnetic bead-based automated platforms are suitable for gDNA extraction from blood samples collected in PAXgene Blood RNA Tubes. Systematic pre-treatments optimization provides guidance for routine and high-throughput workflows, and storage conditions and freeze-thaw cycles offer practical reference for biobanking.

## Introduction

With the development of genomic DNA (gDNA) sequencing techniques, the need for gDNA is increasing in clinical diagnosis and research studies [1]. The gDNA can be extracted from a variety of blood sample types, including blood samples collected in some unconventional tubes [2], [3], [4], [5], [6], [7], [8], [9], [10], [11]. Whole blood collected in PAXgene Blood RNA Tube (referred to as “PAXgene RNA blood samples”) are typically used for RNA extraction. However, when PAXgene RNA blood samples are the only ones available, particularly for clinical studies, they can be used to extract gDNA. Augello et al. [9] developed the PAXgene Blood RNA Tube and evaluated the gDNA extraction from PAXgene RNA blood samples using Qiagen manual kit. Kelly et al. [10] extracted gDNA from 100 µL PAXgene RNA blood samples by using the QIAamp Blood Mini Kit for Short Tandem Repeat (STR) analysis. Kruhoffer et al. [11] used automated platform QIAxtractor with QIAamp DNA Blood Kit from 1 mL PAXgene RNA blood samples; after vacuum concentration, the gDNA samples were analyzed for Single Nucleotide Polymorphisms (SNPs) using GeneChips. All these three groups used silica membrane column-based extraction kits; however, certain procedural details were unclear or inconsistent, which may confuse researchers attempting to extract gDNA from PAXgene RNA Blood samples. Therefore, systematic optimization of sample pre-treatments is essential for obtaining reproducible and high-quality gDNA extraction, not only for silica-based kits but also for platforms based on different techniques.

Silica membrane column-based method and magnetic bead-based method are the two main methods used in commercial DNA extraction kits. To our knowledge, automated platforms for silica membrane column-based extraction have been developed only by Qiagen, whereas most other automated DNA extraction platforms are based on magnetic bead-based technology, including QIAsymphony SP (Qiagen), Maxwell RSC (Promega), KingFisher Apex (Thermo Fisher) and MagNA Pure 24 (Roche). Due to their widespread use and scalability, magnetic bead-based automated platforms provide clear advantages for high-throughput applications.

In this study, we compared three magnetic bead-based automated methods for gDNA extraction. Pre-treatment steps were systematically optimized by investigating the effects of reagents in PAXgene Blood RNA Tube, room temperature incubation time, or centrifugation speed and time. We also observed the impact of freeze-thaw cycles or the storage period of PAXgene RNA blood samples at 4 °C on gDNA yield. Finally, we conducted high-throughput extractions in 96-well format using QIAsymphony SP platform.

## Materials and methods

### Sample

Peripheral whole blood was collected at NYU Langone Hospital under IRB approval (S16-00122) in 18 PAXgene Blood RNA Tubes (PreAnalytix/Qiagen, Hilden, Germany) and three K2-EDTA Tubes (BD Vacutainer, Becton Dickinson, Franklin Lakes, NJ, USA; hereafter referred to as “EDTA blood”). All samples were de-identified according to the protocol and followed HIPAA regulations. PAXgene Blood RNA samples were stored at −80 °C before extraction.

### Pre-treatment of PAXgene RNA blood samples

Since pre-treatment instructions are not provided by the manufacturer, we determined that PAXgene RNA blood samples were thawed and incubated at room temperature for indicated times. After rotating on Tube Revolver Rotator (Thermo Fisher Scientific, Waltham, MA, USA) at speed 10 (about 30 rpm) for 10 min, 1.5 mL of PAXgene RNA blood samples were aliquoted in 1.5 mL EP tubes. A 1.5 mL aliquot of PAXgene RNA blood samples were centrifuged at indicated speed for indicated time, the supernatant was discarded, and the pellet was resuspended with 400 or 450 μL of phosphate buffered saline (PBS, Corning Inc., Corning, NY, USA) by vortex at high speed for 5 min.

### DNA extraction

#### Extract DNA using QIAsymphony SP automated platform

Blood samples or pellet suspensions were extracted using the QIAsymphony SP automated platform (Qiagen, Hilden, Germany) and the commercial DSP DNA Midi Kit (Qiagen, Hilden, Germany) following the manufacturer’s instructions. After pre-treatment, a 450 μL aliquot was transferred to a new vial for loading onto the QIAsymphony SP and extracted using blood extraction program: DNA_Blood_400_V6_DSP (Qiagen, Hilden, Germany). DNA was eluted with 100 μL of elution buffer.

Among the three magnetic bead-based automated platforms evaluated in this study, two supported high-throughput extraction in 96-well format and one supported 48-well format (Table 1). Considering both gDNA quality and yield, the QIAsymphony SP platform was selected for high-throughput extraction. In high-throughput extraction, the pre-treated blood samples were processed in 96-well format by loading 96 samples onto the QIAsymphony SP system in a single batch.

**Table 1:** DNA quality, quantity, and platform characteristics of four DNA extraction methods.

Extraction method (n≥3)	Kit type	Kit chemistry	Available format	DNA concentration, ng/μL mean ± SD	A260/A280 mean ± SD	A260/A230 mean ± SD	DIN value	Main peak^b^, kb
DNeasy blood & tissue kit	Manual	Silica-based	Single tube	43.06 ± 51.31	1.86 ± 0.05	3.68 ± 2.38	8.53 ± 0.06	55.13 ± 6.19
QIAsymphony SP	Automated	Magnetic bead-based	96-well	42.73 ± 21.93	1.79 ± 0.06	1.80 ± 0.85	9.40 ± 0.26	>60
Maxwell RSC	Automated	Magnetic bead-based	48-well	48.52 ± 29.57	1.85 ± 0.06	1.77 ± 0.44	7.60 ± 0.66	17.24 ± 2.98
KingFisher apex	Automated	Magnetic bead-based	96-well	1.36 ± 1.02	1.69 ± 0.54	1.09 ± 0.61	N.A.	>60
EDTA control^a^				107.04 ± 34.19	1.81 ± 0.01	2.83 ± 0.09	8.57 ± 0.50	57.18 ± 3.08

^a^EDTA control gDNA was extracted from EDTA blood using the QIAsymphony SP platform. ^b^Main peak corresponds to the highest-intensity DNA band on the TapeStation electropherogram, reported as molecular weight (kb).

#### Extract DNA using maxwell RSC automated platform

The pellet suspensions were extracted using the Maxwell RSC automated platform (Promega Corporation, Madison, WI, USA) and the commercial Maxwell RSC Whole Blood DNA Kit (Promega Corporation, Madison, WI, USA) following the manufacturer’s instructions. After pre-treatment, a 400 μL of pellet suspension was loaded to the sample well of Maxwell RSC Whole blood DNA Kit and extracted using blood extraction program (Promega Corporation, Madison, WI, USA). DNA was eluted with 100 μL of elution buffer.

#### Extract DNA using KingFisher apex automated platform

The pellet suspensions were extracted using the KingFisher Apex automated platform (Thermo Fisher Scientific, Waltham, MA, USA) and the commercial MagMAX DNA Multi-Sample Ultra 2.0 Kit (Thermo Fisher Scientific, Waltham, MA, USA) following the manufacturer’s instructions. After pre-treatment, a 400 μL of pellet suspension was transferred to deep 96-well plate for loading onto the KingFisher Apex and extracted using blood extraction program MagMAX_Ultra2_400 µL_V2 (Thermo Fisher Scientific, Waltham, MA, USA). DNA was eluted with 100 μL of elution buffer.

#### Extract DNA using Qiagen manual kit

After pre-treatment, a 400 μL of pellet suspension was extracted using DNeasy Blood & Tissue Kit (Qiagen, Hilden, Germany), a silica membrane column-based kit, following the manufacturer’s instructions, and the DNA was eluted with 100 μL of elution buffer.

### DNA quantitation

The concentration and the purity (A260/A280 and A260/A230) of DNA were assessed using NanoDrop Microvolume UV–Vis Spectrophotometer (NanoDrop ONEc, Thermo Fisher Scientific, Waltham, MA, USA). DNA integrity was assessed using agarose gel electrophoresis and also checked on the TapeStation 4,200 System (Agilent, Santa Clara, CA, USA) using Genomic DNA Screentape (#5067–5365) and associated Genomic DNA Reagents (#5067–5366). The DNA Integrity Number (DIN) value and the highest molecular weight of the main band of DNA was determined using the TapeStation 4,200 software.

### Polymerase chain reaction (PCR)

The quality of DNA was assessed by PCR using primers specific to human gDNA, SHGC105883 [12] (Stanford Human Genome Center): forward 5′-GTC​AGA​AGA​CTG​AAA​ACG​AAG​CC-3′ and reverse 5′-GCT​TGC​CAC​ATC​CTT​CTT​CAA​GT-3′, resulting in a PCR product of 295 bp. PCR mixtures contained 12.5 μL of One Taq 2X Master Mix (New England Biolabs Inc., Ipswich, MA, USA), 0.2 μM of each primer (Sigma-Aldrich, St. Louis, MO, USA), 1 μL of DNA (0.95–102 ng) and H_2_O to a final volume of 25 μL. Samples were amplified as follow: 1 cycle at 94 °C for 30 s; 35 cycles with steps at 94 °C for 30 s, at 58 °C for 30 s, at 68 °C for 1 min; and a final cycle at 68 °C for 5 min on a T100 Thermocycler (Bio-Rad Laboratories, Hercules, CA, USA).

### Hematoxylin and eosin staining and imaging

Ethylenediaminetetraacetic acid (EDTA) blood smears or pellet suspension smears from PAXgene RNA blood samples were prepared using standard procedures [13]. The smear slides were stained with H&E [14] and scanned using the Aperio AT2 Whole Slide Scanner (Leica Microsystems, Wetzlar, Germany).

### Statistical analysis

All statistics analyses were conducted using GraphPad Prism 10 (GraphPad Software Inc., San Diego, CA, USA). Statistical analyses were performed using appropriate paired or unpaired tests based on data distribution and study design. Normality was assessed using the Shapiro–Wilk test. Parametric tests included paired t-tests and one-way ANOVA; non-parametric tests included Wilcoxon signed-rank test, Friedman test, and Kruskal–Wallis test. A p-value<0.05 was considered statistically significant.

## Results

### Systematic pre-treatment optimization for gDNA extraction from PAXgene RNA blood samples using the QIAsymphony SP platform

#### The effect of the reagents in PAXgene blood RNA tube on gDNA extraction

**Figure 1:**
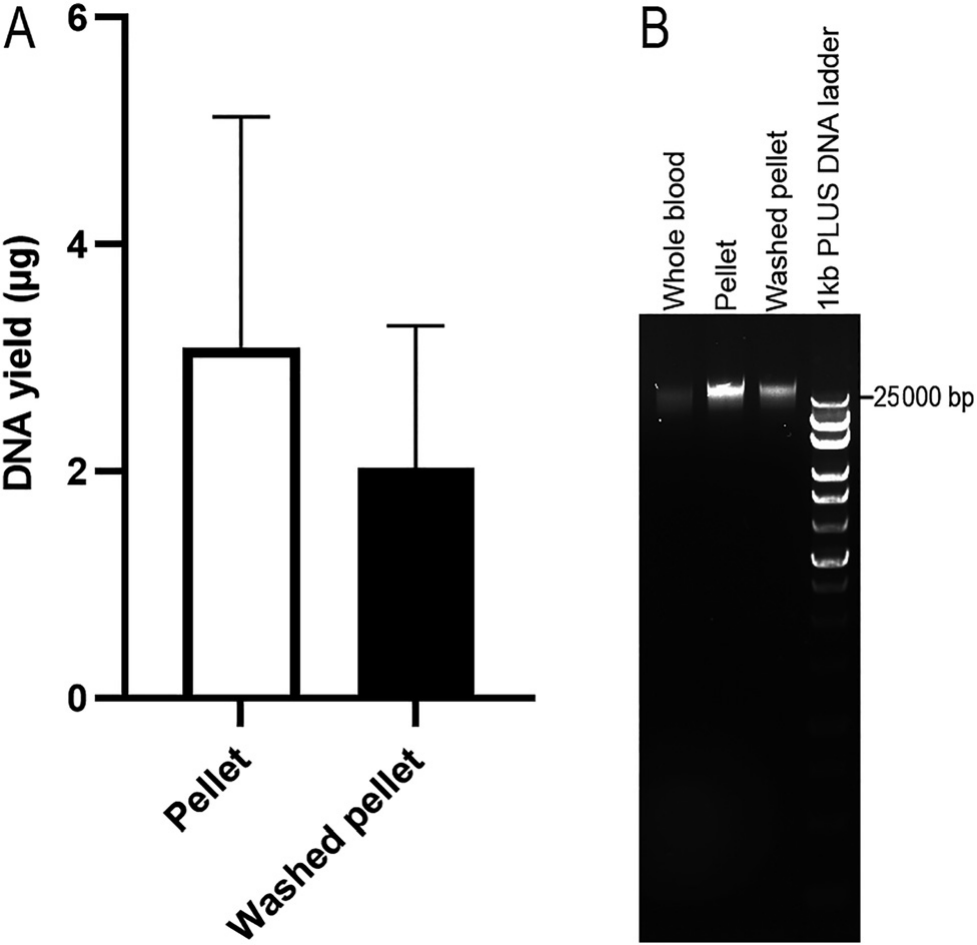
The effect of the reagents in PAXgene blood RNA tube on DNA yield. The gDNA was extracted using the QIAsymphony SP automated platform (n=3, paired samples). DNA yield was assessed using a NanoDrop spectrophotometer (A). Agarose gel electrophoresis of these DNA is shown in (B).

To investigate the effect of reagents in PAXgene Blood RNA Tubes on gDNA extraction, blood samples were thawed and kept at room temperature for 2 h. Then, 450 µL of whole blood was centrifuged at 5,000×*g* for 10 min to collect the pellet. Another 450 µL aliquot of whole blood was centrifuged at 5,000×*g* for 10 min, the pellet was resuspended in 1 mL of PBS, and then centrifuged at 5,000×*g* for 10 min to collect the washed pellet. Both the pellet and the washed pellet were resuspended in 450 µL of PBS, and the suspension was extracted using the QIAsymphony SP platform. These samples were compared to the direct extraction of 450 µL of whole blood from PAXgene Blood RNA Tubes. The gDNA elution from the whole blood samples were brown and could not be measured for concentration by using NanoDrop Microvolume UV–Vis Spectrophotometer. The DNA yield from the pellet suspension was 3.09 ± 2.04 µg (n=3), while the DNA yield from the washed pellet was 2.03 ± 1.25 µg (n=3), with no significant difference between the two groups (paired t-test, p=0.145, Figure 1A). Gel electrophoresis of gDNA (1 μL) revealed clear DNA bands over 25 kb from both the pellet suspension and the washed pellet (Figure 1B). These results indicate that collecting the pellet by centrifugation is necessary, but washing the pellet with PBS is not.

**Figure 2:**
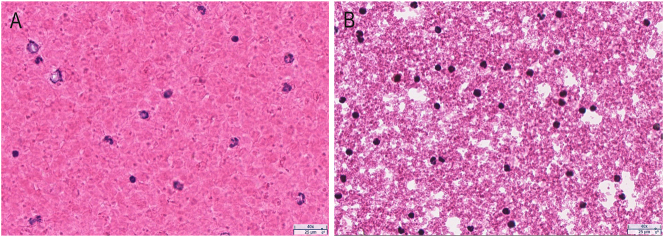
The pellet from PAXgene RNA blood samples (40 × magnification). The smear slides were prepared from EDTA blood (A) and the pellet suspension from PAXgene RNA blood samples (B). After staining with H&E, the slides were scanned using the aperio AT2 whole slide scanner.

The pellet from the PAXgene RNA blood sample was observed (Figure 2B) to contain many cells with intact, morphologically identifiable nuclei (blue), which are the source of gDNA. Compared to EDTA blood (Figure 2A), the cells from PAXgene RNA blood samples are morphologically much smaller.

#### Optimization of centrifugation conditions for gDNA extraction from PAXgene RNA blood samples

The gDNA from 400 µL of EDTA blood is sufficient for whole-genome sequencing [5]. Since 1.5 mL of PAXgene RNA blood sample is equivalent to 400 µL of EDTA blood, this volume was used for method development in this study.

**Figure 3:**
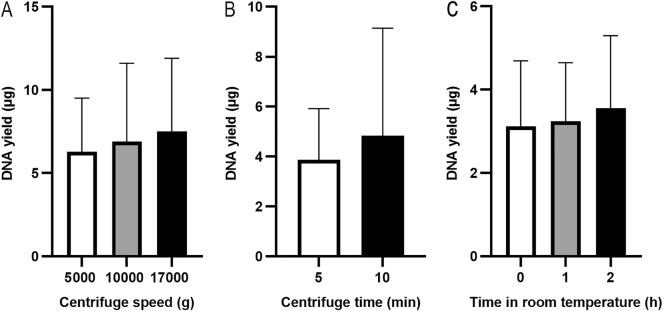
DNA yields from different extraction conditions. Pellets were collected at various centrifuge speeds (A) (n=3, paired samples) and at 17,000×*g* for 5 or 10 min (B) (n=3, paired samples). PAXgene RNA blood samples were thawed and kept at room temperatures for 0, 1 or 2 h (C) (n=3, paired samples). The DNA was extracted using the QIAsymphony SP automated platform, and DNA yield was assessed using a NanoDrop spectrophotometer.

The PAXgene RNA blood samples were thawed and kept at room temperature for 2 h. To investigate the effect of centrifugation speed, 1.5 mL blood aliquots were centrifuged at 5000×*g* [9], 10,000×*g*, or 17,000×*g* for 10 min to collect the pellet. The pellet was resuspended in 450 µL of PBS, and the suspension was extracted using the QIAsymphony SP platform. The DNA yield was 6.27 ± 3.24 μg at 5,000×*g*, 6.90 ± 4.71 μg at 10,000×*g*, and 7.52 ± 4.39 μg at 17,000×*g* (Figure 3A, n=3), with no significant difference (Friedman test, p>0.99). To determine the optimal centrifugation time, 1.5 mL of PAXgene RNA blood samples were centrifuged at 17000×*g* for 5 min or 10 min. The DNA yield was higher at 10 min (4.85 ± 4.29 µg) compared to 5 min (3.87 ± 2.04 µg) with no significant difference (Figure 3B, n=3, Wilcoxon test, p=0.75).

#### The effect of room temperature incubation on gDNA extraction from PAXgene RNA blood samples

The handbook of PAXgene Blood RNA Kit [15] mentioned that after thawing, incubating PAXgene RNA blood samples at room temperature for 2 h before extraction is crucial for RNA extraction. To determine whether this step is also important for gDNA extraction, the PAXgene RNA blood samples were kept at room temperatures for 0, 1, or 2 h before processing. The 1.5 mL of PAXgene RNA blood samples was centrifuged at 17,000×*g* for 10 min. The pellet was resuspended in 450 µL of PBS, and the suspension was extracted using the QIAsymphony SP platform. The DNA yield was 3.12 ± 1.57 µg after the PAXgene RNA blood samples were thawed at room temperature for 0 h, 3.24 ± 1.40 µg for 1 h, and 3.55 ± 1.74 µg for 2 h (Figure 3C, n=3), with no significant difference in DNA yield among the time points (Friedman test, p=0.36).

### gDNA extraction from PAXgene RNA blood samples using four commercial kits

**Figure 4:**
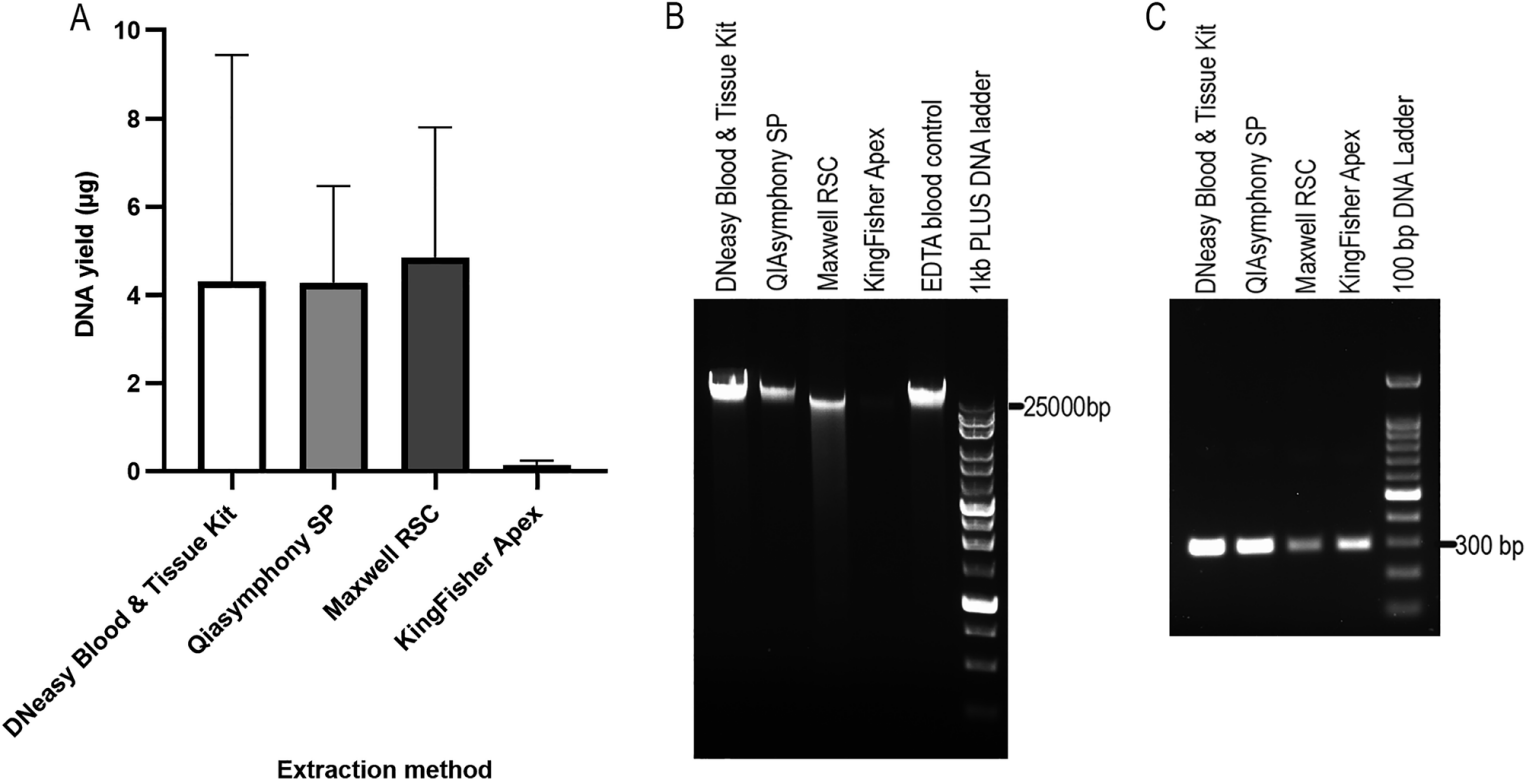
DNA yield and electrophoresis analysis of gDNA from four different extraction methods, as well as their PCR products. DNA yields are shown in (A) (n≥3, unpaired samples due to limited material, kruskal-Wallis, p=0.01). Agarose gel electrophoresis of these gDNA samples are shown in (B), with gDNA extracted from EDTA blood used as control. The PCR products (295 bp) of these gDNA samples are shown in (C).

The PAXgene RNA blood samples were thawed and kept at room temperature for 2 h. Pellets were collected from 1.5 mL of blood samples by centrifugation at 17,000×*g* for 10 min. The pellet was resuspended in 400 or 450 µL of PBS, and gDNA was extracted using four different methods: DNeasy Blood & Tissue Kit (manual control), Qiasymphony SP, Maxwell RSC, and KingFisher Apex (Table 1). EDTA blood extracted with QIAsymphony SP was used as an additional control to evaluate DNA integrity. DNA concentration, A260/A280 and A260/A230 ratios, DIN values, and the main-band molecular weights are shown in Table 1. DNA yields from the four extraction methods are compared in Figure 4A (Kruskal-Wallis, p=0.01). The gDNA sample with the highest yield from each method was selected for gel electrophoresis (Figure 4B). Among the automated magnetic bead-based extraction platforms, QIAsymphony SP produced the highest-quality gDNA: DNA concentration was comparable to the manual kit control, the gel band was the brightest, DIN values exceeded those of EDTA control, and TapeStation analysis showed a main peak over 60 kb (Figure S1). PCR amplification was successful (Figure 4C), confirming suitability for downstream applications. Maxwell RSC yielded gDNA with the highest concentration, slightly above manual kit control, but gel band was less intense and showed smearing (Figure 4B); DIN values and main-band molecular weight were much lower than those of the EDTA control (Figure S1), and PCR bands were relatively faint (Figure 4C). KingFisher Apex produced the lowest DNA yield, insufficient for DIN measurement; however, the main peak was>60 kb (Figure S1), and PCR amplification was successful (Figure 4C). Overall, QIAsymphony SP provides the best combination of yield, integrity, and suitability for downstream applications, while Maxwell RSC produced acceptable results and KingFisher Apex performed poorly.

### The effect of other factors on gDNA extraction

#### The effect of freeze-thaw cycles on gDNA yield from PAXgene RNA blood samples

**Figure 5:**
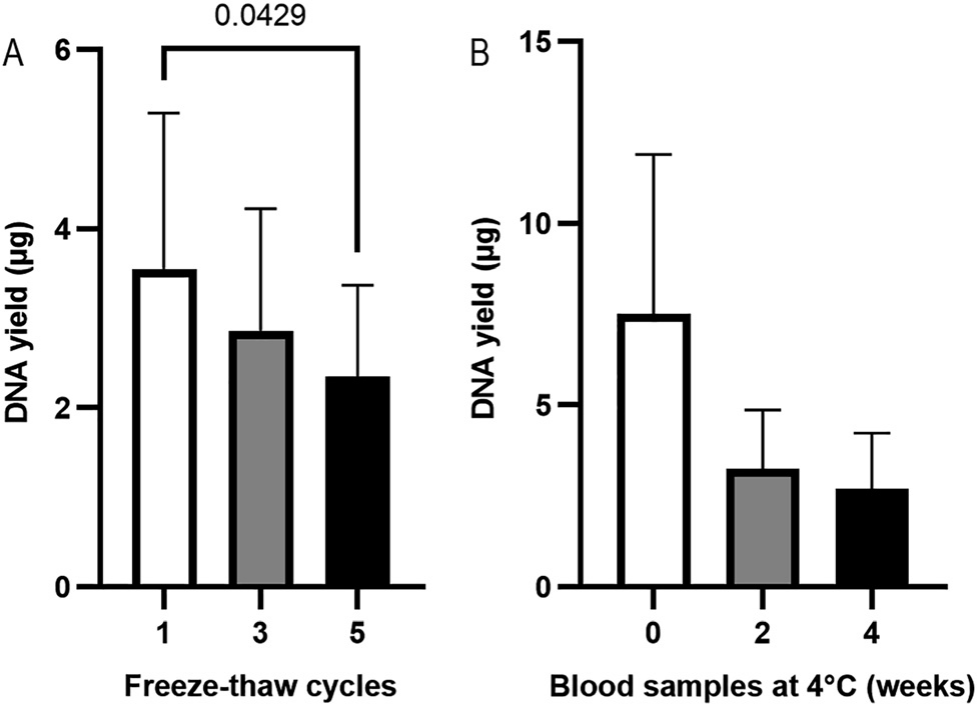
The effect of freeze-thaw cycles (A) (n=3, paired samples, Friedman test, p=0.03) and the storage at 4 °C (B) (n=3, paired samples, Friedman test, p=0.19) of the PAXgene RNA blood samples on DNA yield. gDNA was extracted using the QIAsymphony SP automated platform, and DNA yield was assessed using a NanoDrop spectrophotometer.

PAXgene RNA blood samples can undergo up to two freeze-thaw cycles without affecting RNA yield or stability, according to the product introductions [16]. To investigate the effect of freeze-thaw cycles on gDNA extraction, PAXgene RNA blood samples were applied to 1–5 freeze-thaw cycles (n=3). The samples were then thawed and kept at room temperature for 2 h. Pellets were collected from 1.5 mL of blood samples by centrifugation at 17,000×*g* for 10 min. The pellet was resuspended in 450 µL of PBS, and the suspension was extracted using the QIAsymphony SP platform. As shown in Figure 5A, the highest DNA yield (3.55 ± 1.74 µg) was obtained from samples with 1 freeze-thaw cycle. Friedman test showed a significant difference between the three groups (p=0.03). Pairwise comparisons showed a significant difference between 1 freeze-thaw cycle and 5 freeze-thaw cycles (p=0.04).

#### The effect of storage time at 4 °C on gDNA yield from PAXgene RNA blood samples

Blood samples in PAXgene blood RNA Tubes can be stored at 4 °C for up to 5 days for RNA extraction, according to the product introductions (Qiagen), whereas EDTA blood can be stored at 4 °C for months for DNA extraction [5]. To investigate the effect of storage time at 4 °C on gDNA extraction from blood samples in PAXgene Blood RNA Tubes, 1.5 mL of blood aliquots (n=3) were stored at 4 °C for 0, 2 or 4 weeks (Figure 5B). Pellets were collected from 1.5 mL of blood samples by centrifugation at 17,000×*g* for 10 min. The pellet was resuspended in 450 µL of PBS, and the suspension was extracted using the QIAsymphony SP platform. The DNA yield from PAXgene RNA blood samples stored at 4 °C in 0, 2, 4 weeks was 7.52 ± 4.39, 3.26 ± 1.61 and 2.70 ± 1.53 µg. The highest DNA yield was obtained from blood samples stored for 0 weeks, which was about 2 folds of that of the blood samples stored in 4 °C for 2 weeks, suggesting that storage of the PAXgene RNA blood samples at 4 °C for 2 weeks should be prohibited.

### Extraction of gDNA from PAXgene RNA blood samples in 96-well format

**Figure 6:**
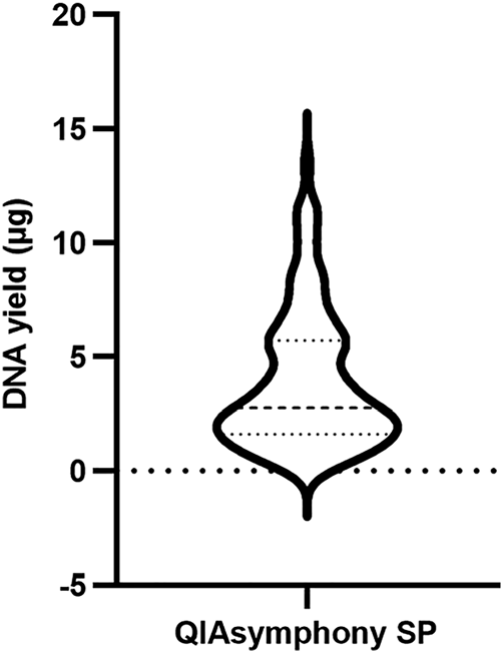
The DNA yield of PAXgene RNA blood samples extracted by QIAsymphony SP in 96-well format (n=96). The median was 2.77 µg, with the 25th and 75th percentile at 1.62 and 5.73 µg, respectively.

To collect additional data from clinical samples and evaluate extraction efficiency in a high-throughput format, PAXgene RNA blood samples were thawed and incubated at room temperature for 2 h. Pellets were collected from 1.5 mL of blood samples by centrifugation at 17,000×*g* for 10 min. The pellet was resuspended in 450 µL of PBS, and the suspension was extracted using the QIAsymphony SP platform in 96-well format. The DNA yield from samples extracted using QIAsymphony SP (n=96) ranged from 0.24 to 13.46 µg, with an average of 3.92 ± 2.99 µg. A total of 12.50 % of samples had DNA yields less than 1 µg, while 66.67 % of them were within the range of 1–6 µg (Figure 6). The median value was 2.77 µg. The average A260/280 was 1.89 ± 0.89. Detailed data for each sample can be found in the Supplementary Material (Supplementary Table S1). All these samples were conducted methylation assay (data not shown), which just required 500 ng DNA [17].

## Discussion

EDTA blood or buffy coat is one of the best sources for gDNA extraction [18]. Although the gDNA yield from PAXgene RNA blood samples (3.92 ± 2.99 µg) in our high-throughput extractions was much lower than the gDNA yield from 400 µL of EDTA blood (30.26 ± 23.93 µg) in our previous study [5], 87.50 % of them still had a concentration above 10 ng/μL in 100 µL, which is adequate for most gDNA sequencing techniques. To our knowledge, observation of the pellet from the PAXgene RNA blood samples has not been reported. According to the product information of PAXgene Blood RNA Tube, the pellet should be lysed cells. However, it was unexpected that, although the cells were shrunken, we still could see intact cells in the pellet (Figure 1B). Many of the cells contained nuclei, which are the source of gDNA, while no nuclei were observed in the supernatant (data not shown).

Previous studies [9], [10], [11] have reported gDNA extraction from PAXgene RNA blood samples; however the specific pre-treatment details and key factors of the blood samples remain unclear or inconsistent, emphasizing the need for systematic optimization. Kelly et al. [10] centrifuged the blood sample at 4 °C, which is not recommended. According to the protocol for RNA extraction from blood samples collected in PAXgene Blood RNA Tube [15], the blood sample should be incubated at room temperature for at least 2 h and then centrifuged at room temperature before RNA extraction. Although the effect of centrifugation temperature on gDNA extraction was not evaluated in our study, it is reasonable to conclude that centrifugation at 4 °C is unnecessarily. Augello et al. [9] and Kelly et al. [10] washed cell pellet with water before DNA extraction; however, according to our results (Figure 1A), this step is unnecessary. In this study, we also investigated the effects of incubation time of blood samples at room temperature or centrifuge conditions. We found that incubating blood samples at room temperature for longer improved DNA yield (Figure 3C), and centrifugation at higher speed for longer time enhanced DNA yield as well (Figure 3A and B). However, there was no significant difference. Usually, it takes at least 1 h at room temperature to fully thaw the whole tube of PAXgene RNA blood samples (approximately 10 mL). After thawing, the whole blood can either be extracted immediately or incubated at room temperature for an additional 2 h to obtain more gDNA. If the whole blood is centrifuged at a lower speed, the pellet will be easier to resuspend. Based on our experimental results, researchers may appropriately adjust pre-treatment conditions to balance processing time and DNA yield, particularly in high-throughput applications using 96-well format.

Currently, only a few studies [9], [10], [11] have extracted gDNA from PAXgene RNA blood samples. All used silica membrane column-based methods, and obtained 2.25 µg [9], 3.75 µg [10] and 2.07 µg [11] of gDNA from 1.5 mL of PAXgene RNA blood samples (normalized), which are lower than the yields obtained with the QIAsymphony SP (4.27 ± 2.19 µg) and Maxwell RSC (4.85 ± 2.96 µg) platform in our study. This difference highlights the potential benefits of magnetic bead-based automated platforms for improving DNA recovery from PAXgene RNA blood samples. Our results indicate that the QIAsymphony SP platform consistently produced the highest-quality gDNA. Although Maxwell RSC yielded high DNA amounts, partial degradation and weaker PCR bands were observed, whereas KingFisher Apex produced significantly lower DNA yields. Notably, these findings suggest that not all magnetic bead-based kits are equally effective for extracting sufficient gDNA from PAXgene RNA blood samples. To date, only the Qiagen manual kit has been reported for gDNA extraction from PAXgene RNA blood samples. This variability suggests the importance of platform-specific validation when using any new DNA extraction kit.

In conclusion, not all magnetic bead-based platforms are suitable for gDNA extraction from PAXgene RNA blood samples. Systematic pre-treatment optimization provides important guidance for routine and high-throughput extraction workflows, and our observations on storage conditions and freeze-thaw cycles offer practical reference for biobanking.

## Supplementary Material

Supplementary Material
