# Supplementary material for: Optimization and comparison of genomic DNA extraction from whole blood collected in PAXgene blood RNA tube using automated platforms
Source: Clin Chem Lab Med. 2025 Oct 20;64(3):600–8. doi: 10.1515/cclm-2025-1079 (PMC12862341; doi:10.1515/cclm-2025-1079)

**Table S1: DNA concentration, yield and quality from PAXgene RNA blood samples**

|  | DNA Concentration, ng/µl | DNA yield, µg | A260/280 |
| --- | --- | --- | --- |
|  | 68.95 | 6.90 | 1.81 |
|  | 112.30 | 11.23 | 1.87 |
|  | 99.04 | 9.90 | 1.86 |
|  | 36.57 | 3.66 | 1.80 |
|  | 55.59 | 5.56 | 1.69 |
|  | 56.39 | 5.64 | 1.70 |
|  | 16.08 | 1.61 | 1.88 |
|  | 29.35 | 2.94 | 1.71 |
|  | 60.52 | 6.05 | 1.69 |
|  | 44.01 | 4.40 | 1.80 |
|  | 19.44 | 1.94 | 1.89 |
|  | 27.84 | 2.78 | 1.80 |
|  | 68.30 | 6.83 | 1.72 |
|  | 134.60 | 13.46 | 1.83 |
|  | 15.23 | 1.52 | 1.73 |
|  | 10.39 | 1.04 | 1.66 |
|  | 14.87 | 1.49 | 1.75 |
|  | 116.00 | 11.60 | 1.84 |
|  | 4.93 | 0.49 | 1.35 |
|  | 39.35 | 3.94 | 1.76 |
|  | 85.91 | 8.59 | 1.73 |
|  | 22.14 | 2.21 | 1.61 |
|  | 11.12 | 1.11 | 1.77 |
|  | 50.87 | 5.09 | 1.80 |
|  | 38.06 | 3.81 | 1.79 |
|  | 23.05 | 2.31 | 1.85 |
|  | 63.06 | 6.31 | 1.48 |
|  | 16.00 | 1.60 | 1.93 |
|  | 57.60 | 5.76 | 1.69 |
|  | 97.60 | 9.76 | 1.58 |
|  | 39.27 | 3.93 | 1.81 |
|  | 85.05 | 8.51 | 1.90 |
|  | 13.79 | 1.38 | 2.06 |
|  | 14.61 | 1.46 | 1.94 |
|  | 44.94 | 4.49 | 2.02 |
|  | 27.29 | 2.73 | 1.81 |
|  | 7.67 | 0.77 | 3.29 |
|  | 57.33 | 5.73 | 1.65 |
|  | 59.82 | 5.98 | 1.57 |
|  | 16.37 | 1.64 | 1.80 |
|  | 86.64 | 8.66 | 1.54 |
|  | 85.33 | 8.53 | 1.87 |
|  | 49.18 | 4.92 | 1.91 |
|  | 18.92 | 1.89 | 1.69 |
|  | 23.56 | 2.36 | 1.56 |
|  | 16.30 | 1.63 | 1.85 |
|  | 34.70 | 3.47 | 1.68 |
|  | 8.23 | 0.82 | 2.36 |
|  | 20.86 | 2.09 | 1.96 |
|  | 24.17 | 2.42 | 2.01 |
|  | 37.83 | 3.78 | 1.72 |
|  | 23.33 | 2.33 | 1.60 |
|  | 11.99 | 1.20 | 1.58 |
|  | 66.40 | 6.64 | 1.75 |
|  | 57.36 | 5.74 | 1.69 |
|  | 5.45 | 0.55 | 2.00 |
|  | 26.27 | 2.63 | 1.77 |
|  | 3.51 | 0.35 | 9.79 |
|  | 106.00 | 10.60 | 1.88 |
|  | 34.61 | 3.46 | 1.84 |
|  | 77.14 | 7.71 | 1.80 |
|  | 32.79 | 3.28 | 1.62 |
|  | 9.93 | 0.99 | 1.50 |
|  | 4.52 | 0.45 | 2.89 |
|  | 2.44 | 0.24 | 4.55 |
|  | 20.14 | 2.01 | 1.57 |
|  | 17.32 | 1.73 | 1.77 |
|  | 12.15 | 1.22 | 1.58 |
|  | 70.06 | 7.01 | 1.75 |
|  | 38.87 | 3.89 | 1.52 |
|  | 79.14 | 7.91 | 1.69 |
|  | 25.52 | 2.55 | 1.72 |
|  | 7.31 | 0.73 | 2.27 |
|  | 21.39 | 2.14 | 1.66 |
|  | 18.68 | 1.87 | 1.71 |
|  | 40.35 | 4.04 | 1.68 |
|  | 57.05 | 5.71 | 1.65 |
|  | 55.86 | 5.59 | 1.69 |
|  | 113.50 | 11.35 | 1.62 |
|  | 8.50 | 0.85 | 2.02 |
|  | 23.68 | 2.37 | 1.67 |
|  | 26.02 | 2.60 | 1.67 |
|  | 26.94 | 2.69 | 1.82 |
|  | 21.88 | 2.19 | 1.78 |
|  | 15.72 | 1.57 | 1.51 |
|  | 51.88 | 5.19 | 1.69 |
|  | 14.57 | 1.46 | 1.75 |
|  | 32.64 | 3.26 | 1.76 |
|  | 29.67 | 2.97 | 1.73 |
|  | 50.63 | 5.06 | 1.77 |
|  | 72.30 | 7.23 | 1.79 |
|  | 6.43 | 0.64 | 1.74 |
|  | 27.59 | 2.76 | 1.61 |
|  | 21.14 | 2.11 | 1.48 |
|  | 6.57 | 0.66 | 1.62 |
|  | 25.19 | 2.52 | 1.81 |
| Average | 39.24 | 3.92 | 1.89 |
| SD | 29.93 | 2.99 | 0.89 |


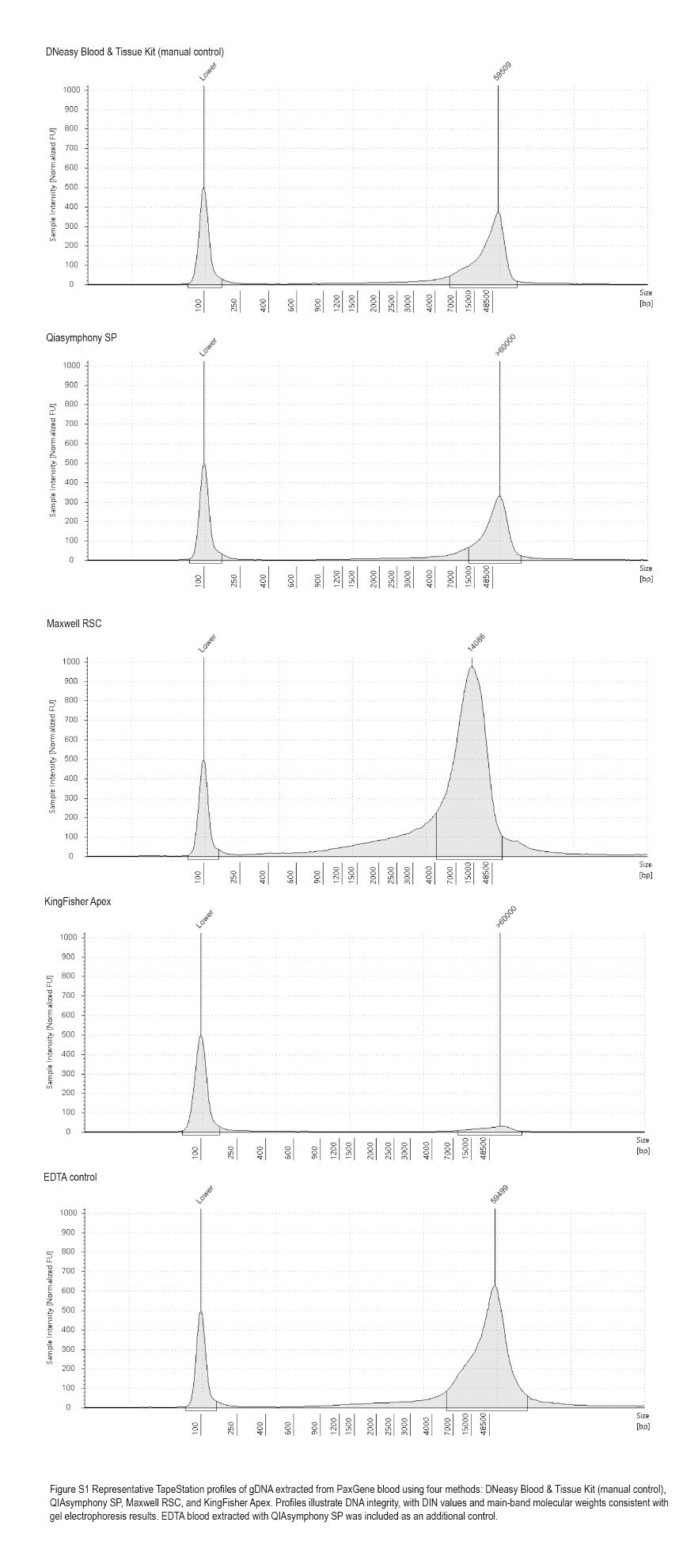

Supplement: Supplementary file 1 — Supplementary Material [file j_cclm-2025-1079_suppl_001.docx]
